# Proteomic Profile Differences in Immune-Related Diseases in Pediatric Patients Under Five Years Old: Asthma and IgE-Dependent Allergies—A Pilot Study

**DOI:** 10.3390/ijms27177769

**Published:** 2026-08-30

**Authors:** Natalia Rzetecka-Mańka, Joanna Matysiak, Eliza Matuszewska-Mach, Paulina Sobkowiak, Irena Wojsyk-Banaszak, Anna Bręborowicz, Paulina Borysewicz, Agnieszka Klupczyńska-Gabryszak, Jan Matysiak

**Affiliations:** 1 Department of Inorganic and Analytical Chemistry, Poznan University of Medical Sciences, 60-806 Poznan, Poland; 2 Doctoral School, Poznan University of Medical Sciences, 60-812 Poznan, Poland; 3 Faculty of Medicine and Health Sciences, University of Kalisz, 62-800 Kalisz, Poland; 4 Department of Pulmonology, Pediatric Allergy and Clinical Immunology, Poznan University of Medical Sciences, 60-572 Poznan, Poland

**Keywords:** asthma, allergy, pediatric, proteomics, protein profiling, STRING

## Abstract

Asthma is a heterogeneous disease that often begins in childhood and frequently occurs alongside allergic conditions. In asthma research, it is important to focus on proteins that are the primary regulators of cellular physiology. The differences in the proteome between children with asthma and those with an atopic background remain poorly understood. The present study included 130 serum samples from four groups of pediatric patients under the age of five: (1) with asthma and IgE-dependent allergies; (2) with non-atopic asthma; (3) non-asthmatics with IgE-dependent allergy; and (4) a control group without asthma and IgE-dependent allergies. The serum samples were used for protein–peptide profiling and proteomic identification using nanoLC-MALDI-TOF/TOF MS/MS. The obtained data were analyzed using univariate statistics and the STRING tool v12.0 to identify protein–protein potential interactions. A total of seven proteins were identified as discriminative between the study groups: A2M, AACT, IgG3, C3, ITIH2, IgG3 and IGK. All of them were upregulated in patients with IgE-dependent allergy compared to other study groups. STRING analysis identified functional associations among four proteins (AACT, A2M, C3, and ITIH2) with discriminatory potential for distinguishing between non-atopic asthma and non-asthmatic patients with IgE-dependent allergy. The results suggest that the identified putative protein markers overlap in cellular pathways, including those associated with the pathophysiology of asthma and allergic disorders. These findings provide further insight into the overall proteomic profile of pediatric patients with asthma and IgE-dependent allergy, highlighting its heterogeneity across the analyzed groups.

## 1. Introduction

Asthma is a common chronic respiratory disease characterized by coughing, wheezing, chest tightness, airway obstruction and shortness of breath [1]. Recent years have seen an increase in the global prevalence and incidence of asthma in children [2,3]. Children exposed to environmental pollution in urban areas, poor-quality housing (allergens), increased obesity rates and stress may be at increased risk of asthma [3]. Asthma, particularly in children under the age of five, remains challenging to diagnose due to different types of asthma phenotypes, as well as the limited ability of young children to express and communicate their symptoms in an effective and coherent way [4]. Furthermore, spirometry is generally not feasible in children under 5 years of age because it requires reliable, reproducible measurements that require active patient cooperation. Spirometry is a standard diagnostic tool in older children and adults. Therefore, the diagnosis of children under five years of age primarily consists of a thorough interview, observation of symptoms, assessment of treatment response, and exclusion of other diseases [4]. If the diagnosis is accurate and made early, the patient not only achieves symptom control more quickly but also has the opportunity for normal psychomotor development.

Asthma is often associated with allergic diseases, the most common phenotype of which is allergic asthma. The best-known proteins involved in controlling the immune response and differentiating asthma phenotypes are cytokines. T2 cytokines (IL-4, IL-5 and IL-13) are signaling proteins produced by type 2 helper T cells that play a crucial role in allergic and eosinophilic responses [5]. They stimulate the production of immunoglobulin E (IgE) and the activation of eosinophils, causing inflammation in asthma.

Despite many proteins already known to play an important role in asthma, there remains a need to identify those that can distinguish the proteomes of asthmatics patients from those with IgE-dependent allergies without asthma under the age of five. The scientific areas focused on exploring differences in protein profiles in asthma include immunology, allergology, molecular biology, and largely through the application of proteomics. Proteomics enables the analysis of changes in protein levels and the study of protein interactions in blood samples, thereby facilitating the identification of new pathophysiological mechanisms in asthma and allergies. The current literature shows that the protein profile changes in childhood asthma and associated conditions [6]. To date, scientific reports on proteomic studies of asthma and allergies in children have primarily focused on other, older age groups, underscoring the need for further research in children under 5 years of age [7,8]. Proteomic analyses enable the detection of molecules present in small quantities in biological samples, such as serum, and the identification of differences in protein profiles. Proteomic analyses employing MALDI-MS and LC-MS/MS facilitate the identification of low-abundance proteins in biological samples, while immunoassays such as ELISA, Western blot, and flow cytometry can be utilized for result validation and quantification. Such studies require sensitive, advanced techniques and could be the next step in identifying new potential indicators of childhood asthma and IgE-dependent allergies or in improving our understanding of the molecular mechanisms of these diseases.

This pilot study aimed to identify differences in the protein profiles in the serum of patients under the age of five with non-atopic asthma (As), asthma and IgE-dependent allergies (As+Al), non-asthmatics with IgE-dependent allergy (Al), and controls (C). Particular attention was paid to selecting study groups that focus on populations underrepresented in the existing literature-children under five years of age. The pilot study employed MALDI-TOF/TOF-MS to identify differences in peptide–protein profiles among the selected patient groups and ELISA to quantitatively confirm the results. Additionally, STRING analysis revealed functional associations and interactions among the identified proteins. The results obtained provide a better understanding of the proteomic basis of asthma and allergic disease pathogenesis and also highlight potential areas for further research into the interactions between the analyzed proteins.

## 2. Results

The performed MALDI-TOF MS proteomic analyses enabled us to detect 209 unique peaks across all samples. The study revealed that intensities of 70 of these precursor ions showed statistically significant differences. In the statistical analysis, pairwise comparisons were conducted between all groups, yielding a total of six comparisons (As vs. As+Al; As vs. Al; As vs. C; As+Al vs. Al; As+Al vs. C; Al vs. C). Statistical analysis based on intensity values identified significant differences in the levels of seven proteins across the investigated groups (Table 1). These proteins were: alpha-1-antichymotrypsin (AACT), alpha-2-macroglobulin (A2M), complement C3 (C3), immunoglobulin G3 (IgG3), herpesvirus entry mediator (HVEM), immunoglobulin kappa (IgK), and inter-alpha-trypsin inhibitor heavy chain 2 (ITIH2) (Table 1). The results were assessed for differences using univariate statistical tests between the following groups of patients: with asthma and IgE-dependent allergy (As+Al), with non-atopic asthma (As), non-asthmatics with IgE-dependent allergy (Al) and control patients (C) (Table 2 and Table S1). We found significant differences between the patient groups As+Al vs. Al and As vs. Al. As presented in Table 1, the levels of proteins in the Al group were higher compared to the As+Al and As groups. No differences were identified between the study groups and the control group. In addition, the Benjamini–Hochberg correction was applied, and all studied proteins had *p*-values below 0.05. The ELISA method was used for quantitative verification (Table S2). Due to very low concentration values below the lower limit of quantitation (ITIH2 (4.7 ng/mL), IgK (3.0 ng/mL), HVEM (0.02 ng/mL) protein kits), these data were not included in further statistical analyses. In each ELISA assay, variability in sample measurements was observed, with some values falling outside the standard curve’s range. These out-of-range values were excluded from further analysis. Consequently, only the AACT, A2M, C3, and IgG3 assays were included in the univariate statistical analysis. The statistical analysis revealed no significant differences between the study groups, with the only statistically significant difference occurring in C3 between the groups Al and C (Table S3).

**Figure 1:**
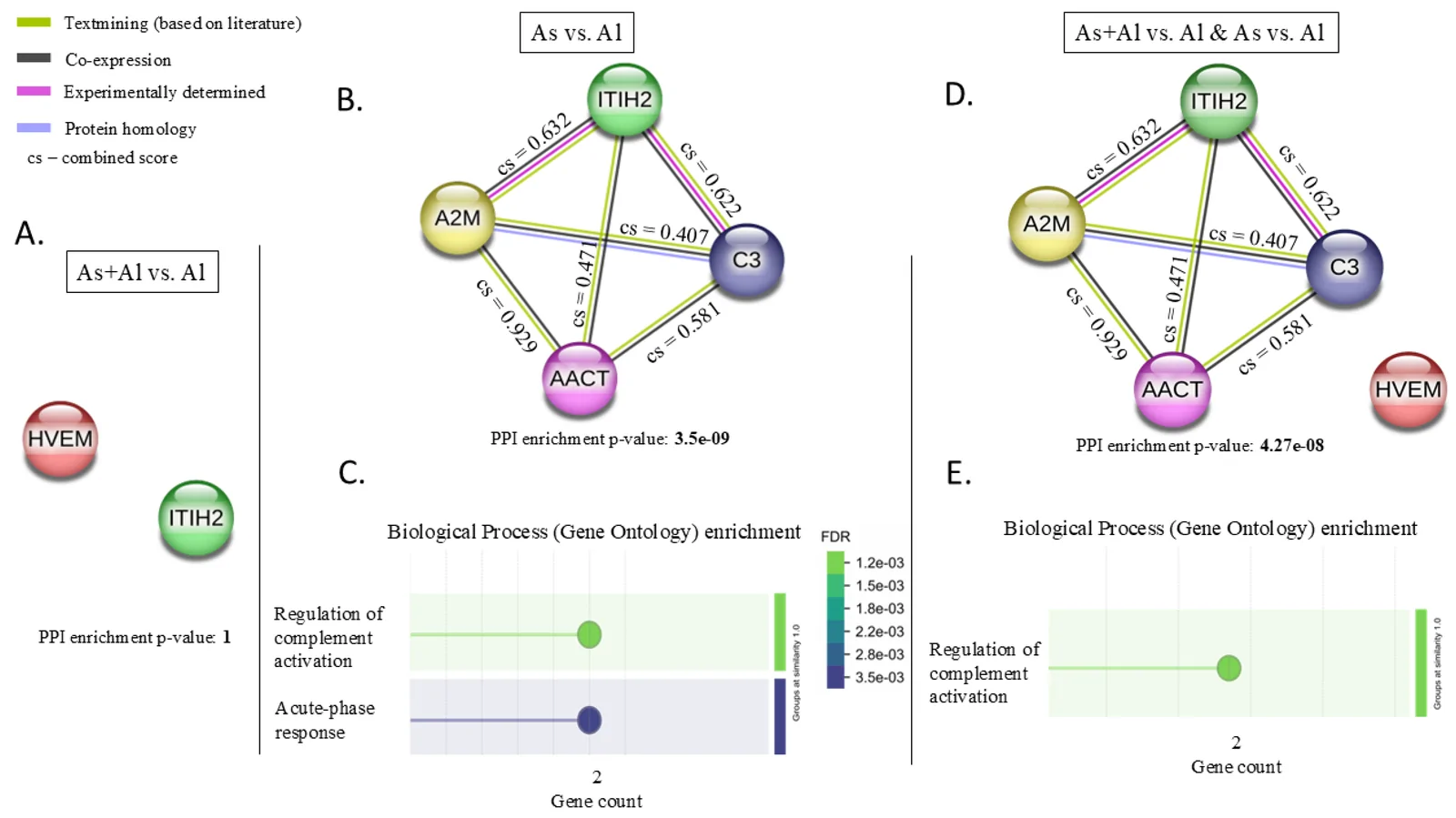
An analysis of functional protein interaction networks conducted using the STRING database for proteins that differentiate the studied groups. The panels (**A**,**B**,**D**) show the networks of functional interactions/connections for the individual protein sets, highlighting differences between the analyzed groups. (**A**) The correlation of the HVEM and ITIH2 proteins for the compared groups As+Al vs. Al, achieving a PPI enrichment *p*-value of 1. (**B**) The correlation of the A2M, ITIH2, AACT and C3 proteins for the compared groups As vs. Al, achieving a PPI enrichment *p*-value of 3.5 × 10^−9^. (**C**) The correlation of the A2M, ITIH2, AACT, C3 and HVEM proteins for the As+Al vs. Al and As vs. Al groups, achieving a PPI enrichment *p*-value of 4.27 × 10^−8^. (**C**,**E**) present the results of the functional enrichment analysis (Biological Process), showing the biological processes in the analyzed protein sets. This demonstrates that the proteins under study are involved in regulation complement activation and the acute phase response. The FDR (false discovery rate) value indicates that the lower the value, the more statistically significant it is that these proteins occur in the same biological context. Abbreviations: As+As, asthma with IgE-dependent allergy; As, non-atopic asthma; Al, non-asthmatics with IgE-dependent allergy. AACT, alpha-1-antichymotrypsin; A2M, alpha-2-macroglobulin; C3, complement C3; ITIH2, inter-alpha-trypsin inhibitor heavy chain 2; cs, combined score.

Peptide–protein profiling identified proteins that were significantly different only between the As+Al vs. Al and As vs. Al groups. Asthma and IgE-dependent allergies are closely related conditions, with IgE-dependent immune responses playing a key role in the development and progression of allergic asthma. Therefore, the next step was to combine the identified proteins and examine their correlations. The STRING version 12.0 program was used to investigate the correlation [9]. We examined protein correlations between the As+Al and Al groups (HVEM, ITIH2) and between the As and Al groups (A2M, ITIH2, AACT, C3). Neither IGK nor IgG3 can be used in the program (Figure 1). We also examined the correlations across all identified proteins (Figure 1).

The combined score (cs) indicates the strength of the association between the proteins (Figure 1). The closer the combined score is to 1, the stronger the association between the proteins. The colors of protein–protein connections indicate specific interactions. Green indicates that the interaction has been confirmed in the literature databases; black indicates co-expression; pink indicates experimentally determined interactions between proteins and blue indicates protein homology. The PPI enrichment *p*-value indicates whether a combination of proteins interact with each other more frequently than would be expected by chance.

Starting with the As+Al vs. Al groups, we identified HVEM and ITIH2 as differentially expressed proteins. STRING analysis showed no correlation between them (PPI enrichment *p*-value = 1) (Figure 1A). The proteins that differed between the As and Al groups were A2M, ITIH2, C3, and AACT (Figure 1B). As asthma often co-occurs with allergies, the underlying proteomic-level features of these conditions are being investigated. In our study, we identified four proteins that distinguish between the two conditions and that may be involved in the various inflammatory processes occurring in both. The connection between A2M, ITIH2, C3, and AACT yielded a PPI enrichment *p*-value of 3.5 × 10^−9^, indicating that these proteins are not randomly associated but rather share a common biological context. In addition, the cs of 0.929 for AACT and A2M indicates that the strongest association among the studied proteins suggests a high probability of functional interaction within shared biological processes. High correlation coefficients were also observed between ITIH2 vs. A2M and ITIH2 vs. C3, with values of 0.632 and 0.622, respectively. By contrast, the associations between ACT and C3 (cs = 0.581) and between A2M and C3 (cs = 0.407) are moderate but still biologically important. The next parameter is the false discovery rate (FDR). The lower the FDR value (0.003) in the acute phase response, the more statistically significant it is that the proteins share a biological context (Figure 1C). The gene count (Figure 1C) indicates the number of proteins included in a given term. Terms are grouped based on their similarity, as measured by the Jaccard index [9]. In our case, this shows that ‘acute phase response’ (blue) is distinct from ‘regulation of complement activation’ (green) (Figure 1D). During the acute phase, A2M and AACT are involved, and A2M and C3 play a role in regulating complement activation.

Then we compared all identified proteins, A2M, ITIH2, C3, AACT and HVEM (Figure 1D,E). Including the HVEM protein (which has a different function from the others) reduced dataset consistency and altered the results of the enrichment analysis, even though the biological core is similar. This is due to the small size of the analyzed dataset and the presence of a protein with a distinct functional profile, which affects the statistical evaluation of overrepresented biological processes. As a receptor involved in immune signaling, HVEM is not involved in acute phase response processes. These results highlight the importance of interpreting enrichment analyses in the context of general biological trends while accounting for the impact of individual elements on the model’s overall functioning. But still A2M, ITIH2, C3, AACT and HVEM achieved a PPI enrichment *p*-value of 4.27 × 10^−8^, also indicating that these proteins are not randomly associated. However, adding HVEM has skewed the analysis towards one side of the process, as this profile has become more significant in the ‘regulation of complement activation’. Including the additional protein HVEM in the set containing four proteins altered the functional enrichment profile, most likely due to the small set size and overlap in Gene Ontology (GO) terms. The results of both analyses should be interpreted as indicating a related biological context rather than completely different functions of the analyzed proteins. Consequently, although these proteins enable discrimination between individual groups such as asthma, IgE-dependent allergy with asthma, and allergy only, when analyzed together, particularly between the As and Al groups (A2M, AACT, C3, ITIH2), they may form a potential biological model reflecting shared and biologically relevant mechanisms.

## 3. Discussion

Asthma and IgE-dependent allergy are closely related conditions, both characterized by airway inflammation and respiratory symptoms, and contribute to the wide spectrum of clinical manifestations and disease progression observed, particularly in children under five years of age. IgE is the most well-known protein involved in allergic reactions [10]. As complex as allergic reactions are, many more proteins are at play, whose roles and involvement still need to be established.

**Figure 2:**
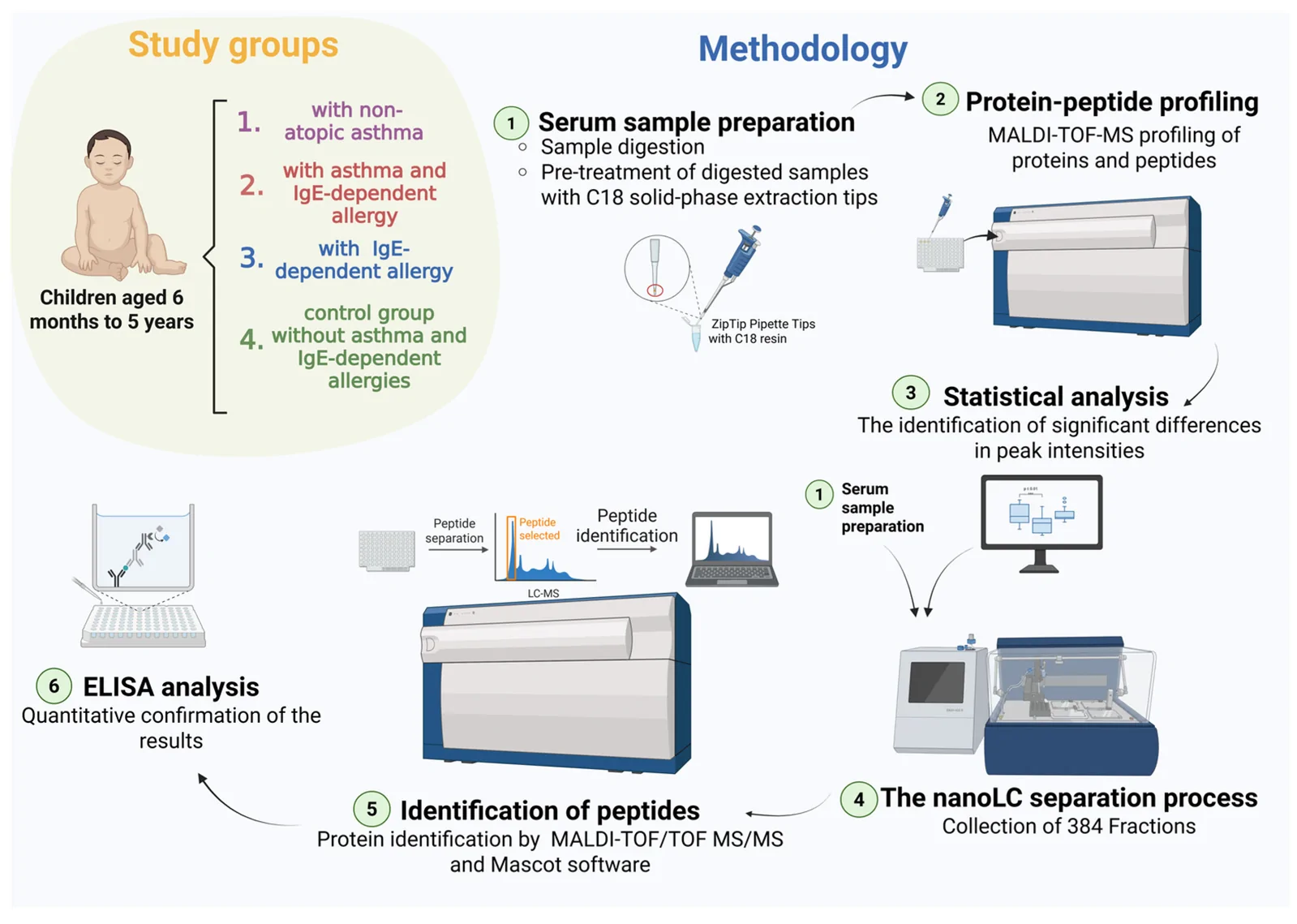
Workflow of the study. Created in BioRender.com.

The objective of this study was twofold: firstly, to investigate differences in protein–peptide profiles among four pediatric groups and secondly, to find interactions between serum proteins discriminating the study groups (non-atopic asthma and non-asthmatics with IgE-dependent allergy) (Figure 2). A key strength of our study is the inclusion of the following distinct study groups: pediatric patients with asthma and an IgE-dependent allergy (As+Al), non-atopic asthma (As), non-asthmatics with IgE-dependent allergy (Al), and a control group (C) consisting of children with neither asthma nor an IgE-dependent allergy. We included these patient groups because asthma often occurs alongside allergies. Another strength of this study is the matching of the groups for age, gender, and sample size (Table 2). This study went beyond the traditional search for univariate indicators, focusing instead on constructing the protein interaction-based model from the identified serum proteins. This enables the integration of biological information from multiple proteins, thereby better reflecting the complexity of pathophysiological processes.

In the present study, differences were observed among the As+Al, As, and Al groups in the signals for the A2M, AACT, C3, ITIH2, and HVEM proteins (Table 1). It is evident that each of these proteins exerts a direct or indirect effect on the mechanisms underlying asthma and allergies. C3 is one of the most important components of the complement system, a fundamental part of the innate immune system that plays a vital role in defending the body against pathogens and in inflammatory processes. In asthma, it likely acts by inducing and/or amplifying the inflammatory response within the complement cascade [11]. A2M is one of the major plasma proteins and plays an important role in hemostasis regulation [12]. Due to its cage-like structure, A2M inhibits protein proteases by trapping them without directly inhibiting their enzymatic activity. The synthesis of A2M is regulated by pro-inflammatory cytokines in both acute and chronic inflammatory diseases [12]. AACT—encoded by the *SERPINE3* gene [13]—is a serine protease inhibitor that plays a role in the acute phase response, inflammation, and proteolysis. ITIH2 belongs to the inter-alpha-trypsin inhibitor (ITI) family, which constitutes a group of serine protease inhibitors in plasma. These consist of a light chain and five homologous heavy chains (HC1-5) [14]. During acute inflammation, the H2 chain is downregulated, while the associated molecules act as acute negative phase proteins [15]. HVEM, also known as TNFRSF14 [16], is a cell membrane-bound receptor that activates the NF-κB pathway, thereby inducing pro-inflammatory and cell survival-promoting genes. It can also act as an immune ‘switch’, performing various activating or inhibitory functions [17].

In our study, all identified proteins were elevated in non-asthmatic patients with IgE-dependent allergy compared to those in the non-atopic asthma and asthma with IgE-dependent allergy groups. The literature shows that these four proteins (A2M, AACT, C3, and ITIH2) are involved in the underlying mechanisms of asthma and allergy. A study by Vedel-Krogh et al. [18] showed that higher plasma C3 levels are associated with a greater risk of hospitalization for asthma and more frequent asthma exacerbations. Fattah et al. [19] reached similar conclusions, demonstrating that C3 levels were significantly higher in 120 pediatric patients with asthma than in healthy children. Nakano et al. [20] showed that plasma C3a levels were significantly higher in patients with severe asthma exacerbations than in control subjects with stable asthma. These studies demonstrate elevated C3 levels during asthma exacerbations and when asthma patients are compared to healthy individuals. Our study shows a stronger C3 signal in patients with IgE-dependent allergies than in those with non-atopic asthma, suggesting a stronger immune response. In a study of patients with stable chronic obstructive pulmonary disease (COPD), Xiao et al. [21] showed that plasma A2M levels were lower than those in healthy controls. In the present study, patients with allergies exhibited higher A2M levels than those with asthma. Zhou et al. [22] found higher AACT levels in asthma patients, but significant differences were observed between those experiencing acute exacerbations and those in clinical remission. This suggests that inflammation exacerbation leads to increased AACT protein levels. Hollander et al. [23] also investigated AACT levels in patients with COPD and found that plasma AACT levels were elevated in COPD patients compared to the control group, but not in patients with genetic AAT deficiency. Our study confirms previously reported findings: elevated AACT levels in patients with allergies. Xiong et al. [24] demonstrated that HVEM expression is elevated in adult patients with severe or moderate persistent asthma compared with those with mild persistent asthma and healthy subjects. Many studies have shown that the levels of the proteins we identified should be higher in patients with asthma than in the control group. However, our research indicates that protein levels (A2M, AACT, C3, ITIH2, and HVEM) were higher in the Al group than in the As group. This suggests increased inflammation and immune responses in IgE-dependent allergy.

Analysis of the STRING network indicates that these proteins are functionally linked, with the strongest relationship observed between AACT and A2M. Also, A2M and C3 belong to the TEP (thioester-bond-containing protein) family [25]. In this context, it is essential to interpret these proteins not only as distinct compounds with differential potential but also as components of a shared biological system associated with inflammatory processes and the immune response. AACT and A2M are associated with the acute phase response and protease regulation, while C3 is linked to the innate immune axis and complement activation [8,26,27]. Studies show that A2M and AACT can undergo proteomic changes in response to various pathological conditions [28]. Okano et al. [26] identified AACT as a potential marker of COPD. Okada et al. [27] investigated proteomic differences between patients with inflammatory bowel disease (IBD) and healthy controls. The study suggests that C3 and A2M proteins may serve as potential markers for IBD to monitor disease progression. According to the literature C3 is associated with asthma, playing a role in its development and severity [19,29]. The prevalence of elevated C3 has been identified in both adults and children diagnosed with asthma and allergic diseases [30]. In our study, we identified the C3 protein as a distinguishing factor between allergic IgE-dependent and asthma, with elevated levels observed in allergies. Going further, ITIH2 has been identified in recent years as a potential biomarker or early predictor of COVID-19 [31,32]. This may indicate its involvement in inflammatory processes in the human body.

As the literature indicates, all of the identified discriminating proteins play a role in the body’s immune processes. However, not all of them have been definitively identified as playing an active role in asthma and allergies. Our research shows that the signals of these proteins differ between groups of children under five years of age. No previous proteomic studies have indicated that A2M, AACT, ITIH2 or C3 are significant in differentiating asthma from allergies in pediatric patients. This is particularly important because the proteomic differences between asthma and IgE-dependent allergy are still being investigated, especially given that these diseases can co-occur [33]. Current advances also include the study of molecules circulating in patients’ serum, such as extracellular vesicles (EVs) [34]. The term “EV” is an umbrella term that encompasses exosomes and microvesicles. They can carry a variety of molecules, including proteins, lipids, metabolites, and microRNAs (miRNAs). The primary focus of research in this area is miRNA in EVs, as miRNA regulates gene expression at the post-transcriptional level and helps control cellular pathways that are often involved in inflammatory processes [34,35]. These studies found that miRNAs are potential biomarkers of the presence and severity of atopic diseases. Although our study focused on proteomic changes, combining both approaches could improve the discovery and characterization of molecular markers in asthma and allergic diseases.

A potential limitation of this study is the restriction of high-resolution analysis in reflector mode to low-molecular-weight peptides. As a result, MS/MS analysis in reflector mode can be applied in the *m*/*z* range 700–3500. As a result, peaks above 3500 *m*/*z* cannot be identified. Moreover, due to the ELISA assay’s limited sensitivity, ITIH2, HVEM, and IgK could not be detected in serum samples from pediatric patients. Another limitation is that the study only considers one time point. Including at least two time points would allow us to determine whether the specific proteins change over time across the four study groups. Additionally, increasing the size of the study groups would strengthen the statistical basis of the analysis.

The next step in research on pediatric non-atopic asthma and allergy IgE-dependent should be to further investigate the proteins identified in this study (A2M, AACT, C3, ITIH2, and HVEM), particularly through expanded proteomic analyses in children younger than 5 years. Further research should include a broader proteomic analysis using methods with higher sensitivity and wider proteome coverage than those applied in the present study. This may enable the identification of additional molecules that differentiate the allergic and asthmatic groups under investigation. The next interesting step may also be to examine the proteomic differences between asthma and diabetes as they exhibit a partly causative association [36,37]. In addition to diabetes, other conditions that could be investigated include obesity and other atopic diseases, such as atopic dermatitis. Taking this approach would improve our understanding of the underlying mechanisms of these diseases and help us to identify their common features and differences. Overall, our study represents an important first step toward further research into differences in the proteomic profiles of pediatric patients.

## 4. Materials and Methods

### 4.1. Sample Collection and Patient Characteristics

Participants were recruited in 2023 and continued until mid-2024 at the Department of Pediatric Pneumonology, Allergology and Clinical Immunology, K. Jonscher Clinical Hospital, Poznan University of Medical Sciences, and Joanna Matysiak Medical Practice in Kalisz. The study was approved by the Bioethics Committee of Poznań Medical University, Poland (Decision No. 960/22), and was conducted in accordance with the Declaration of Helsinki [38]. Prior to sample collection, all parents or legal guardians of the young participants were fully informed about the study and provided written consent.

The study was conducted on serum samples from four groups of subjects aged between 6 months and 5 years: study groups (38 children with asthma and IgE-dependent allergy (As+Al), 35 children with non-atopic asthma (As) and 24 non-asthmatic children with IgE-dependent allergy (Al)) and one control group (33 children without IgE-dependent allergy and asthma (C)) (Figure 2, Table 2). The study group included patients with symptoms of bronchial asthma with or without allergy and patients with allergy without asthma. Asthma diagnosis was made by the referring physician based on the GINA (2022) criteria [4]. Patients with recurrent respiratory infections without allergy and with no signs of bronchial asthma were included in the control group. In patients’ blood smears, total IgE, IgM, IgG, and IgA concentrations were determined, and IgE antibodies to 30 selected allergens were measured using the Atopic Polycheck 30-I panel (Biocheck, Münster, Germany). Between sample collection and proteomic analyses, the samples were stored at −80 °C.

### 4.2. Sample Preparation

The serum samples were prepared according to our modified protocol [39]. The serum samples were diluted with water 60 times and then digested with trypsin from the Pierce modified trypsin digestion kit (Promega, Madison, WI, USA). To a clean 500 µL tube, 15 µL of Digestion Buffer (50 mM ammonium bicarbonate in ultrapure water) and 1.5 µL of Reducing Buffer (100 mM dithiothreitol in ultrapure water) were added. Then, 10.5 µL of the protein sample was added to the mixture, which was vortexed and incubated at 95 °C for 5 min. Afterward, 20 µL of Alkylation Buffer (100 mM iodoacetamide in ultrapure water) was introduced, and the samples were incubated for 20 min in the dark at room temperature. The samples were then incubated at 37 °C for up to 16 h, with the addition of 2 µL of trypsin. After the digestion time, the reaction was stopped by adding 3 µL of 10% trifluoroacetic acid (TFA) to the tubes.

The next step was to purify, desalt, and concentrate the digested samples using a ZipTip C18 reverse-phase chromatography pipette tip (Millipore, Bedford, MA, USA). ZipTip C18 tips were conditioned with acetonitrile (ACN) and equilibrated with 0.1% TFA in water. For maximum protein binding, 10 µL of the sample solution was aspirated and dispensed 10 times. Next, the pipette tip used was washed with 0.1% TFA, and the bound proteins and peptides on the C18 column were eluted with 5 µL of 50% ACN in 0.1% TFA.

### 4.3. Protein–Peptide Profiling

A matrix solution was prepared according to the HCCA protocol (Bruker Daltonics, Bremen, Germany). A total of 0.7 mg/mL α-cyano-4-hydroxycinnamic acid (HCCA) was dissolved in 85% ACN, 15% H_2_O, 0.1% TFA, and 1mM NH_4_H_2_PO_4_. A total of 750 nL of prepared samples were spotted on the AnchorChip 384 target plate in three replicates. After the plate had dried, 1 µL of the prepared matrix solution was applied spot-on to the dried sample droplet. For nanoLC fractions a matrix solution was prepared containing 748 µL TA95 (95 ACN: 5 TFA 0.1% in water), 36 µL saturated HCCA solution in 90 ACN: 15 TFA 0.1% in water, 8 µL TFA 10% in water, and 8 µL 100 mM NH_4_H_2_PO_4_. For external calibrants, a matrix solution was prepared containing 748 µL TA85 (85 ACN: 15 TFA 0.1% in water), 36 µL saturated HCCA solution in 90 ACN: 15 TFA 0.1% in water, 8 µL TFA 10% in water, and 8 µL 100 mM NH_4_H_2_PO_4_. The matrix for the external calibrant solution was mixed in 300 µL with 1.5 µL of the peptide calibration standard II (peptide calibration standard II dissolved in 125 µL (30 ACN: 70 TFA 0.1% in water)) and spotted 420nl of this mixture on each of the calibrant positions on the AnchorChip 384 target plate (Bruker Daltonics, Bremen, Germany) manually. The plate with dried droplets was analyzed using an UltrafleXtreme MALDI-TOF mass spectrometer (Bruker Daltonics, Bremen, Germany) in reflectron-positive mode across an *m*/*z* range of 700–3500 [39]. In a MALDI-TOF/TOF mass spectrometer, ions are separated based on their *m*/*z* ratio, which determines their flight time through the analyzer. The data were pre-processed in the R programming environment (RStudio v2024.12.0+467) and then statistically analyzed 4.6 Statistical analysis), and, among all mass-to-charge ratios (*m*/*z*), those with statistically significant differences in signal intensity were selected. Those statistically significant *m*/*z* features were then subjected to nanoLC-MALDI-TOF/TOF MS/MS proteomic identification.

### 4.4. Proteomic Identification Using nanoLC-MALDI-TOF/TOF MS/MS

The next step involved identifying proteins based on statistically significant differences in signal intensity after protein–peptide profiling. Ten digested serum samples purified with ZipTip C18 tips were combined and processed according to the previously described protocol (Section 4.2). The sample matrix solution for nanoLC fractions and the corresponding calibration solution were prepared (Section 4.3). The separation and collection of 384 fractions were performed using a nano-liquid chromatography (EASY-nLC II) and Proteineer-fc II collector of fractions (Bruker Daltonics, Bremen, Germany). Our previous research provides a comprehensive account of the MS analysis procedure, including pertinent conditions and parameters [39,40].

The target plate was analyzed on MS/MS mode using an UltrafleXtreme mass spectrometer (Bruker Daltonics, Bremen, Germany). The MS/MS technique allowed the identification of proteins and peptides in the *m*/*z* range 700–3500, in the reflectron mode. In MALDI-TOF mass spectrometry, *m*/*z* is the fundamental parameter used for ion separation and detection, enabling the identification of peptides and proteins by their molecular masses and the masses of their fragments. WARP-LC 1.3 software (Bruker Daltonics, Bremen, Germany) was used to identify the precursor ion list, and FlexControl 3.4, FlexAnalysis 3.4, and BioTools 3.2 software (Bruker Daltonics, Bremen, Germany) were used to acquire, process, and evaluate the spectra. The SwissProt and NCBInr database and the Mascot 2.4.1 search engine, taxonomically restricted to *Homo sapiens*, were used to identify discriminative proteins and peptides.

### 4.5. ELISA

The ELISA method was used to confirm the semi-quantitative results of the identified proteins (Assaypro LLC, 3400 Harry S Truman Blvd St. Charles, MO, USA; ELK Biotechnology Co., Ltd., 10410 Corporate Dr, STE12 Sugar Land, TX, USA). ELISA analyses were performed according to the protocols.

### 4.6. Data Analysis

After protein–peptide profiling, the data were reviewed, and the average of the intensity across six replicates per sample was calculated. *m*/*z* features with more than 30% missing data were rejected. Missing values comprising less than 30% of the dataset for a given *m*/*z* feature were replaced with half the minimum sample value. After data cleaning, the obtained dataset was subjected to univariate statistical analysis using PQStat software 2025 (PQStat v.1.8.6.126, Poznan, Poland). The normality of the data distribution was assessed using the Shapiro–Wilk test. Then, Levene’s test was used to assess homogeneity of variance across two or more groups. Finally, the *t*-test or Mann–Whitney U test was performed. A value of *p* < 0.05 was considered statistically significant. In addition, the analysis used the Benjamini–Hochberg correction for multiple comparisons. Following protein identification, a univariate analysis of ELISA data was performed using the appropriate test (*t*-test or Mann–Whitney U test) depending on the results of the Shapiro–Wilk and Levene tests, as previously described (Table S3). Also, we used the STRING database (Search Tool for the Retrieval of Interacting Genes/Proteins) [41]. This database contains data on various types of protein–protein interactions.

## 5. Conclusions

The study was conducted to identify differences in protein profiles among pediatric patients with non-atopic asthma, non-asthmatics with IgE-dependent allergy, asthma with IgE-dependent allergy, and controls. Differences in protein profiles were observed among the study groups, particularly between the asthma and the IgE-dependent allergy group. The findings of the present study demonstrate that, even among diseases that share a similar inflammatory mechanism, pediatric patients exhibit differences in protein–peptide profiles. This study demonstrates that the proteins A2M, AACT, C3, and IHIT2 play a role in both the complement system and the acute phase response. Therefore, it is suggested that the current results be referred to as “phenotype-associated candidates”. The research provides a foundation for further studies aimed at identifying differences in the mechanisms of allergic diseases, particularly in patients under five years of age with non-atopic asthma and non-asthmatics with IgE-dependent allergy.

## Figures and Tables

**Table 1:** Proteins discriminative between study groups identified based on *m*/*z* values using MALDI-TOF MS.

Proteins	HVEM Human	A2M Human	IGK Human	ITIH2 Human	ITIH2 Human	AACT Human	IgG3 Human	Complement C3 Human
**Groups**	**As+Al ↓** vs. **Al ↑**	**As ↓** vs. **Al ↑**	**As+Al ↓** vs. **Al ↑**	**As ↓** vs. **Al ↑**	**As+Al ↓** vs. **Al ↑**	**As ↓** vs. **Al ↑**	**As ↓** vs. **Al ↑**	**As ↓** vs. **Al ↑**
**Mass-to-charge ratio (*m*/*z*):**	1347.737	1621.804	1798.891	1804.892	1804.892	1901.988	1921.966	2445.263
***p*-value**	0.0226	0.0411	0.0047	0.0002	0.0418	0.0007	0.0291	0.0324
***p-value (Benjamini–Hochberg)***	0.0418	0.0418	0.0125	0.0016	0.0418	0.0028	0.0418	0.0418

Abbreviations: As+Al, asthma with IgE-dependent allergy; As, non-atopic asthma; Al, non-asthmatics with IgE-dependent allergy; AACT, alpha-1-antichymotrypsin; A2M, alpha-2-macroglobulin; C3, complement C3; IgG3, immunoglobulin G3; HVEM, herpesvirus entry mediator; IGK, immunoglobulin kappa; ITIH2, inter-alpha-trypsin inhibitor heavy chain 2; ↓, downregulated; ↑, upregulated.

**Table 2:** Characteristics of the patient groups (protein–peptide profiling).

	Patients with Asthma and IgE-Dependent Allergy	Patients with Non-Atopic Asthma	Non-Asthmatic Patients with IgE-Dependent Allergy	Control Patients Without Asthma and IgE-Dependent Allergy
*No. of subjects*	38	35	24	33
*Female*	14	14	8	14
*Male*	24	21	16	19
*Age (year)*				
*Median*	3.38	3.08	3.75	2.83
*Mean ± SD*	3.18 ± 1.04	2.98 ± 1.29	3.72 ± 0.93	2.98 ± 1.44
*Range*	1.33–4.92	0.75–4.92	1.25–4.92	0.58–4.92
*Average concentration of immunoglobulin*				
*IgA (g/L)*	0.63	0.53	0.70	0.63
*IgM (g/L)*	0.87	0.72	0.82	0.81
*IgG (g/L)*	7.96	7.64	8.18	6.94
*IgE (kU/I)*	158.68	47.92	97.06	30.68
*Allergy*	100%	0%	100%	0%
*Allergy classes based on IgE concentration (**%**)*				
*Class 2*	94.74		79.17	
*Class 3*	36.84		50.00	
*Class 4*	15.79		20.83	
*Class 5*	7.89		8.33	
*Class 6*	5.26		4.17	

## Data Availability

The data that support the findings of this study are available from the corresponding author upon reasonable request.

## Supplementary Materials

The following supporting information can be downloaded at: https://www.mdpi.com/article/10.3390/ijms27177769/s1.

## References

[1] Zhou W., Tang J. (2025). Prevalence and Risk Factors for Childhood Asthma: A Systematic Review and Meta-Analysis. BMC Pediatr..

[2] Caffrey Osvald E., Bower H., Lundholm C., Larsson H., Brew B.K., Almqvist C. (2020). Asthma and All-Cause Mortality in Children and Young Adults: A Population-Based Study. Thorax.

[3] Grant T.L., Wood R.A. (2022). The Influence of Urban Exposures and Residence on Childhood Asthma. Pediatr. Allergy Immunol..

[4] Global Initiative for Asthma. https://ginasthma.org/.

[5] Rzetecka N., Matysiak J., Matysiak J., Sobkowiak P., Wojsyk-Banaszak I., Bręborowicz A., Packi K., Klupczyńska-Gabryszak A. (2025). Metabolomics in Childhood Asthma—A Promising Tool to Meet Various Clinical Needs. Curr. Allergy Asthma Rep..

[6] Asamoah K., Chung K.F., Kermani N.Z., Bodinier B., Dahlen S.-E., Djukanovic R., Bhavsar P.K., Adcock I.M., Vuckovic D., Chadeau-Hyam M. (2024). Proteomic Signatures of Eosinophilic and Neutrophilic Asthma from Serum and Sputum. eBioMedicine.

[7] Ding H., Shi Z., Lin H., Sun Y., Zhang L., Liu F., Wang X., Zhang Z., Zhang G. (2024). Serum Proteomics Identifies Novel Diagnostic Biomarkers for Asthma in Preschool Children. J. Thorac. Dis..

[8] Wu Y.-Q., Cai Y.-X., Chen X.-L., Chen S.-Q., Huang X.-F., Lin Z.-L. (2024). Proteomic Analysis Reveals Potential Therapeutic Targets for Childhood Asthma through Mendelian Randomization. Clin. Transl. Allergy.

[9] Szklarczyk D., Nastou K., Koutrouli M., Kirsch R., Mehryary F., Hachilif R., Hu D., Peluso M.E., Huang Q., Fang T. (2024). The STRING Database in 2025: Protein Networks with Directionality of Regulation. Nucleic Acids Res..

[10] Rasheed Z., Zedan K., Saif G.B., Salama R.H., Salem T., Ahmed A.A., El-Moniem A.A., Elkholy M., Al Robaee A.A., Alzolibani A.A. (2018). Markers of Atopic Dermatitis, Allergic Rhinitis and Bronchial Asthma in Pediatric Patients: Correlation with Filaggrin, Eosinophil Major Basic Protein and Immunoglobulin E. Clin. Mol. Allergy.

[11] Zhang X., Köhl J. (2010). A Complex Role for Complement in Allergic Asthma. Expert Rev. Clin. Immunol..

[12] Lagrange J., Lecompte T., Knopp T., Lacolley P., Regnault V. (2022). Alpha-2-Macroglobulin in Hemostasis and Thrombosis: An Underestimated Old Double-Edged Sword. J. Thromb. Haemost..

[13] Lara-Velazquez M., Zarco N., Carrano A., Phillipps J., Norton E.S., Schiapparelli P., Al-kharboosh R., Rincon-Torroella J., Jeanneret S., Corona T. (2021). Alpha 1-Antichymotrypsin Contributes to Stem Cell Characteristics and Enhances Tumorigenicity of Glioblastoma. Neuro-Oncol..

[14] Jiang J., Zhao J., Wang Y., Liu D., Zhang M. (2022). Urine Inter-Alpha-Trypsin Inhibitor Family-Related Proteins May Serve as Biomarkers for Disease Activity of Lupus. J. Clin. Lab. Anal..

[15] Zhang X., Zhang X., Guo L., Bai Y., Tian Y., Luo H. (2024). The Function of the Inter-Alpha-Trypsin Inhibitors in the Development of Disease. Front. Med..

[16] Steinberg M.W., Cheung T.C., Ware C.F. (2011). The Signaling Networks of the Herpesvirus Entry Mediator (TNFRSF14) in Immune Regulation. Immunol. Rev..

[17] Shui J.-W., Steinberg M.W., Kronenberg M. (2011). Regulation of Inflammation, Autoimmunity, and Infection Immunity by HVEM-BTLA Signaling. J. Leukoc. Biol..

[18] Vedel-Krogh S., Rasmussen K.L., Nordestgaard B.G., Nielsen S.F. (2021). Complement C3 and Allergic Asthma: A Cohort Study of the General Population. Eur. Respir. J..

[19] Fattah M.A., El Baz M., Sherif A., Adel A. (2010). Complement Components (C3, C4) as Inflammatory Markers in Asthma. Indian J. Pediatr..

[20] Nakano Y., Morita S., Kawamoto A., Suda T., Chida K., Nakamura H. (2003). Elevated Complement C3a in Plasma from Patients with Severe Acute Asthma. Respir. Dis..

[21] Xiao X., Cai W., Ding Z., Shi Y., Fan L., Zhang Q. (2023). A2M Serves as Promising Biomarker for Chronic Obstructive Pulmonary Disease. Int. J. Chron. Obstruct. Pulmon. Dis..

[22] Zhou Y., Kuai S., Pan R., Li Q., Zhang J., Gu X., Ren H., Cui Y. (2023). Quantitative Proteomics Profiling of Plasma from Children with Asthma. Int. Immunopharmacol..

[23] Hollander C., Westin U., Wallmark A., Piitulainen E., Sveger T., Janciauskiene S.M. (2007). Plasma Levels of Alpha1-Antichymotrypsin and Secretory Leukocyte Proteinase Inhibitor in Healthy and Chronic Obstructive Pulmonary Disease (COPD) Subjects with and without Severe A1-Antitrypsin Deficiency. BMC Pulm. Med..

[24] Xiong Y., Li B., Zhang Y., Shi F., Qiu C., Wang L., Wang J., Le Y., Du Y., Yao C. (2021). Expression of Herpesvirus Entry Mediator Gene as a Potential Biomarker for Disease Severity in Patients with Persistent Asthma. J. Asthma.

[25] Nonaka M., Anderluh G., Gilbert R. (2014). Evolution of the Complement System. MACPF/CDC Proteins—Agents of Defence, Attack and Invasion.

[26] Okano T., Seike M., Kuribayashi H., Soeno C., Ishii T., Kida K., Gemma A. (2016). Identification of Haptoglobin Peptide as a Novel Serum Biomarker for Lung Squamous Cell Carcinoma by Serum Proteome and Peptidome Profiling. Int. J. Oncol..

[27] Okada K., Itoh H., Ikemoto M. (2021). Serum Complement C3 and A2-Macroglobulin Are Potentially Useful Biomarkers for Inflammatory Bowel Disease Patients. Heliyon.

[28] Jin Y., Wang W., Wang Q., Zhang Y., Zahid K.R., Raza U., Gong Y. (2022). Alpha-1-Antichymotrypsin as a Novel Biomarker for Diagnosis, Prognosis, and Therapy Prediction in Human Diseases. Cancer Cell Int..

[29] Najam F.I.E., Shembesh A.H., Giasuddin A.S.M. (2005). Complement Components (C3, C4) in Childhood Asthma. Indian J. Pediatr..

[30] Inoue H., Mashimo Y., Funamizu M., Shimojo N., Hasegawa K., Hirota T., Doi S., Kameda M., Miyatake A., Kohno Y. (2008). Association Study of the C3 Gene with Adult and Childhood Asthma. J. Hum. Genet..

[31] Jørgensen A.G., Dupont D.M., Fjelstrup S., Bus C., Hansen C.B., Benfield T., Garred P., Heegaard P.M.H., Kjems J. (2024). Unbiased Plasma Profiling Using Pre-Selected RNA Aptamer Pools Predicts Mortality in COVID-19 and Identifies Protein Risk Factors. Mol. Ther. Nucleic Acids.

[32] Sahin A.T., Yurtseven A., Dadmand S., Ozcan G., Akarlar B.A., Kucuk N.E.O., Senturk A., Ergonul O., Can F., Tuncbag N. (2022). Plasma Proteomics Identify Potential Severity Biomarkers from COVID-19 Associated Network. Proteom. Clin. Appl..

[33] Yue M., Tao S., Gaietto K., Chen W. (2024). Omics Approaches in Asthma Research: Challenges and Opportunities. Chin. Med. J. Pulm. Crit. Care Med..

[34] Alashkar Alhamwe B., Potaczek D.P., Miethe S., Alhamdan F., Hintz L., Magomedov A., Garn H. (2021). Extracellular Vesicles and Asthma—More Than Just a Co-Existence. Int. J. Mol. Sci..

[35] Grueso-Navarro E., Navarro P., Laserna-Mendieta E.J., Lucendo A.J., Arias-González L. (2023). Blood-Based Biomarkers for Eosinophilic Esophagitis and Concomitant Atopic Diseases: A Look into the Potential of Extracellular Vesicles. Int. J. Mol. Sci..

[36] Mubanga M., Gong T., Smew A.I., Wikström A., Caffrey Osvald E., Eeg-Olofsson K., Janson C., Lundholm C., Almqvist C. (2025). Association between Asthma and Type 2 Diabetes in a Swedish Adult Population: A Register-Based Cross-Sectional Study. Thorax.

[37] Uppal P., Mohammed S.A., Rajashekar S., Giri Ravindran S., Kakarla M., Ausaja Gambo M., Yousri Salama M., Haidar Ismail N., Tavalla P., Hamid P. (2023). Type 2 Diabetes Mellitus and Asthma: Pathomechanisms of Their Association and Clinical Implications. Cureus.

[38] Sawicka-Gutaj N., Gruszczyński D., Guzik P., Mostowska A., Walkowiak J. (2022). Publication Ethics of Human Studies in the Light of the Declaration of Helsinki—A Mini-Review. J. Med. Sci..

[39] Jaskiewicz-Rajewicz K., Wysocka A., Matuszewska-Mach E., Rzetecka N., Maleszka-Kurpiel M., Wozniak J., Michalski A., Udziela M., Szaflik J.P., Ploski R. (2026). Common and Distinct Features in Serum Proteomic Profiles in Keratoconus, Post-Laser Vision Correction Ectasia, and Pellucid Marginal Degeneration. Investig. Ophthalmol. Vis. Sci..

[40] Matuszewska E., Matysiak J., Rosiński G., Kędzia E., Ząbek W., Zawadziński J., Matysiak J. (2021). Mining the Royal Jelly Proteins: Combinatorial Hexapeptide Ligand Library Significantly Improves the MS-Based Proteomic Identification in Complex Biological Samples. Molecules.

[41] Szklarczyk D., Kirsch R., Koutrouli M., Nastou K., Mehryary F., Hachilif R., Gable A.L., Fang T., Doncheva N.T., Pyysalo S. (2023). The STRING Database in 2023: Protein-Protein Association Networks and Functional Enrichment Analyses for Any Sequenced Genome of Interest. Nucleic Acids Res..

