# Supplementary material for: Proteomic Profile Differences in Immune-Related Diseases in Pediatric Patients Under Five Years Old: Asthma and IgE-Dependent Allergies—A Pilot Study
Source: Int J Mol Sci. 2026 Aug 30;27(17):7769. doi: 10.3390/ijms27177769 (PMC13566050; doi:10.3390/ijms27177769)
Supplement: Supplementary file 1 [file ijms-27-07769-s001.zip › ijms-4415867-supplementary.pdf]

Table S1. Characteristics of the patient groups (ELISA).

	Patients with asthma and IgE-dependent allergy	Patients with non-atopic asthma	*Non-asthmatics patients with IgE-dependent allergy*	Control patients without asthma and IgE-dependent allergy
No. of subjects	10	12	13	11
Female	4	4	5	5
Male	6	8	8	6
Age (year)				
Median	2.875	2.21	4.00	3.1
Mean ± SD	3.02±0.98	2.36±1.06	3.8±0.91	3.35±1.26
Range	1.3-4.5	1.0-4.17	1.92-4.92	1.58-4.92
Average concentration of immunoglobulin				
IgA (g/L)	0.632	0.45	0.68	0.67
IgM (g/L)	0.892	0.66	0.91	0.66
IgG (g/L)	8.637	7.02	8.02	7.07
IgE (kU/I)	41.12	39.85	69.65	37.05
Allergy				
Allergy classes based on IgE concentration (%)				
Class 2	80		69	
Class 3	20		46	
Class 4	10		15	
Class 5	0		0	
Class 6	0		0	

Table S2 Protein concentration values measured by ELISA.

**Parameters**	**Concentration (mg/mL)**			
	**IgG3**	**ACT**	**C3**	**A2M**
**As+Al**				
Average ± SD	0.540 ± 0.089	0.293 ± 0.062	1.812 ± 0.062	3.531 ± 0.062
Median	0.563	0.301	1.703	2.253
Q1-Q3	0.493-0.595	0.236-0.313	1.705-2.001	2.306-3.382
**As**				
Average ± SD	0.527 ± 0.149	0.329 ± 0.114	1.792 ± 0.288	3.649 ± 0.837
Median	0.491	0.301	1.912	3.642
Q1-Q3	0.456-0.601	0.266-0.358	1.640-1.980	3.044-4.026
**Al**				
Average ± SD	0.539 ± 0.106	0.297 ± 0.054	1.606 ± 0.309	3.161 ± 0.688

Median	0.523	0.317	1.712	3.165
Q1-Q3	0.4765-0.558	0.291-0.331	1.553-1.767	2.551-3.812
**C**				
Average ± SD	0.470 ± 0.061	0.329 ± 0.067	1.881 ± 0.197	3.671 ± 0.809
Median	0.449	0.297	1.858	3.808
Q1-Q3	0.433-0.518	0.284-0.356	1.732-2.027	2.860-4.267

Abbreviations: As+Al, asthma with IgE-dependent allergy; As, non-atopic asthma; Al, non-asthmatics with IgE-dependent allergy; C, control without asthma and IgE-dependent allergy ; AACT, alpha-1-antichymotrypsin; A2M, alpha-2-macroglobulin; C3, complement C3; IgG3, immunoglobulin G3.

Table S3. P-value for IgG3, AACT, C3, A2M from ELISA analysis.

**Compared groups**	**p-value**			
	**IgG3**	**AACT**	**C3**	**A2M**
**As+Al vs As**	0.796 [1]	0.448 [2]	0.863[1]	0.137 [2]
**Al vs As**	0.815 [1]	0.849 [2]	0.086 [2]	0.123 [1]
**As+Al vs Al**	0.964 [1]	0.598 [2]	0.128 [2]	0.514 [2]
**As+Al vs C**	0.086 [1]	0.277 [2]	0.767 [1]	0.067 [2]
**Al vs C**	0.119 [1]	0.608 [2]	0.027 [2]	0.057 [2]
**As vs C**	0.364 [1]	0.531 [2]	0.632 [1]	0.369 [2]

Abbreviations: As+Al, asthma with IgE-dependent allergy; As, non-atopic asthma; Al, non-asthmatics with IgE-dependent allergy; C, control without asthma and IgE-dependent allergy; AACT, alpha-1-antichymotrypsin; A2M, alpha-2-macroglobulin; C3, complement C3; IgG3, immunoglobulin G3; [1] t-test, [2] Mann-Whitney
